# SUFFERING BUT SATISFIED: OLDER ADULTS’ LIFE SATISFACTION WITHSTANDS DEPRESSION AND DEMENTIA

**DOI:** 10.1093/geroni/igac059.2205

**Published:** 2022-12-20

**Authors:** Samuel Van Vleet, Danica Bass, Yenifer Morales Mejia, Jeanné Dube, Brandee McKinney, Kailee Prentice, Logan Guillory, Michael Barnett

**Affiliations:** Miami University, Oxford, Ohio, United States; The University of Texas at Tyler, Tyler, Texas, United States; The University of Texas at Tyler, Tyler, Texas, United States; The University of Texas at Tyler, Tyler, Texas, United States; The University of Texas at Tyler, Tyler, Texas, United States; The University of Texas at Tyler, Tyler, Texas, United States; The University of Texas at Tyler, Tyler, Texas, United States; The University of Texas at Tyler, Tyler, Texas, United States

## Abstract

Among older adults with neurocognitive disorders, lower life satisfaction has been linked with poorer aspects of physical and mental health that affect quality of life, including depression. However, these associations between life satisfaction and mental health problems have not been found universally. In this study, we further investigated life satisfaction and depression within clinical and healthy older adult populations. Older adults (N = 98), ages 60 to 90 years either with (n = 25) or without (n = 73) a neurocognitive diagnosis, were administered self-report questionnaires measuring depression and life satisfaction. Results showed that although depression was negatively correlated with life satisfaction among both samples, older adults with dementia had higher depression scores but equivalent life satisfaction scores as older adults without dementia. In fact, both the clinical and healthy samples of older adults averaged on the positive or satisfied half of the life satisfaction scale. Older adults with neurocognitive disorders may generally experience higher depressive symptoms, yet despite challenges to quality of life, they seem to find satisfaction in their daily lives. This may stem from older adults viewing current depressive symptoms as a relatively short-term problem while evaluating life satisfaction across their whole lives. The best protective factors for older adults suffering from dementia and depression may therefore be found both in their present and in their past.

